# Preoperative Anesthesia Virtual Video Consultations in a Preadmission Clinic: Quality Improvement Study

**DOI:** 10.2196/57541

**Published:** 2024-07-25

**Authors:** Yamini Subramani, Jill Querney, Priyanka Singh, Yifan Zhang, Lee-Anne Fochesato, Nida Fatima, Natasha Wood, Mahesh Nagappa

**Affiliations:** 1 Department of Anesthesia and Perioperative Medicine London Health Sciences Centre, St. Joseph Health Care, Schulich School of Medicine and Dentistry Western University London, ON Canada; 2 Department of Nursing London Health Sciences Centre Western University London, ON Canada

**Keywords:** preoperative evaluation, preadmission clinic, telemedicine, remote, virtual care, remote consultation, video consultation, telehealth, online health, digital health, perioperative medicine, preoperative, eMedicine, surgery, consultation, safety, assessment, virtual care, workflow, implementation, integration, hospital

## Abstract

**Background:**

The preadmission clinic (PAC) is crucial in perioperative care, offering evaluations, education, and patient optimization before surgical procedures. During the COVID-19 pandemic, the PAC adapted by implementing telephone visits due to a lack of infrastructure for video consultations. While the pandemic significantly increased the use of virtual care, including video appointments as an alternative to in-person consultations, our PAC had not used video consultations for preoperative assessments.

**Objective:**

This study aimed to develop, implement, and integrate preoperative video consultations into the PAC workflow.

**Methods:**

A prospective quality improvement project was undertaken using the Plan-Do-Study-Act (PDSA) methodology. The project focused on developing, implementing, and integrating virtual video consultations at London Health Sciences Centre and St. Joseph Health Care (London, Ontario, Canada) in the PAC. Data were systematically collected to monitor the number of patients undergoing video consultations, address patient flow concerns, and increase the percentage of video consultations. Communication between the PAC, surgeon offices, and patients was analyzed for continuous improvement. Technological challenges were addressed, and procedures were streamlined to facilitate video calls on appointment days.

**Results:**

The PAC team, which includes professionals from medicine, anesthesia, nursing, pharmacy, occupational therapy, and physiotherapy, offers preoperative evaluation and education to surgical patients, conducting approximately 8000 consultations annually across 3 hospital locations. Following the initial PDSA cycles, the interventions consistently improved the video consultation utilization rate to 17%, indicating positive progress. With the onset of PDSA cycle 3, there was a notable surge to a 29% utilization rate in the early phase. This upward trend continued, culminating in a 38% utilization rate of virtual video consultations in the later stages of the cycle. This heightened level was consistently maintained throughout 2023, highlighting the sustained success of our interventions.

**Conclusions:**

The quality improvement process significantly enhanced the institution’s preoperative video consultation workflow. By understanding the complexities within the PAC, strategic interventions were made to integrate video consultations without compromising efficiency, morale, or safety. This project highlights the potential for transformative improvements in health care delivery through the thoughtful integration of virtual care technologies.

## Introduction

Amid the COVID-19 pandemic, many in-person consultations in the preadmission clinic (PAC) at our tertiary academic centers of London Health Sciences Centre (LHSC) and St. Joseph’s Health Care in London, Ontario, Canada, shifted to telephone consultations. Telephone consultations were instrumental in reducing unnecessary hospital visits and in-person interactions, thereby mitigating the risk of COVID-19 transmission. While phone consultations facilitate thorough patient history–taking and chart review, they inherently lack the capability for a physical examination, which is essential in preanesthesia evaluations. Specifically, an airway assessment, which is critical for anesthesia planning, cannot be conducted effectively over the phone. By integrating a telemedicine model that includes audio and visual components in the PAC, several significant advantages emerge, including (1) an enhanced physical assessment, as the visual capability over video calls ensures a more accurate and comprehensive evaluation than phone consultations; (2) improved patient interaction given that nonverbal communication plays a crucial role in interpreting patient concerns and responses, which is lost in phone consultations; (3) increased diagnostic accuracy, since visual examinations can aid in identifying physical signs that might indicate underlying health issues, which may not be apparent through phone calls; and (4) enhanced patient engagement and education, as visual tools can be used to educate patients about their procedure and anesthesia plan, making it easier for them to understand complex information [1,2].

Telehealth involves electronic video communication between patients and health care providers to improve patient health remotely [3,4]. While telemedicine has long been used in rural areas without access to specialists, its prevalence increased widely during the COVID-19 pandemic [5,6]. When strategically deployed, virtual care enhances the quality and effectiveness of patient care and enables dynamic risk stratification through big data and machine learning [7].

LHSC and St. Joseph’s Health Care collectively handle approximately 50,000 surgical cases annually across various subspecialties. The PAC is a designated setting for multidisciplinary preoperative assessments and optimization of operating room efficiency. Notably, not all patients receive preoperative assessments in the PAC, as limitations in time, office space, and human resources restrict the number of patients seen. The PAC team, comprising professionals from medicine, anesthesia, nursing, pharmacy, occupational therapy, and physiotherapy, offers preoperative evaluation and education to surgical patients, totaling approximately 8000 consultations annually across 3 hospital locations.

Over the years, the PAC has undergone alterations in office location, size, caseload, and staffing. The PAC team’s preoperative consultations often include internal medicine and/or anesthesiology consultations and cover all surgical subspecialties. Some consultations are time-sensitive or involve mandatory in-person visits due to combined procedures such as x-rays, electrocardiograms, echocardiography, surgical team consultations, and blood work. Therefore, implementing video consultations requires meticulous planning and decision-making to ensure smooth clinic operations [8].

On a national and global level, virtual care video appointments have become a popular alternative to in-person and phone appointments during the COVID-19 pandemic [9,10]. Patients benefit from time and cost savings, increased communication with providers, improved access to care, and involvement of family members or caregivers [1,11]. Telemedicine has been shown to reduce missed appointments, wait times, and readmissions; enhance office efficiency with fewer front desk phone calls; and increase medication adherence. The ability of health care providers to make eye contact, assess body language, discuss sensitive topics, and conduct a limited physical examination over a virtual video platform can improve the patient-physician relationship [12]. This approach aligns with the trend toward digital health care solutions and ensures that patient safety and care quality are maintained at the highest standards.

During the COVID-19 pandemic, the PAC adapted by implementing telephone visits due to a lack of infrastructure for video consultations. While the pandemic significantly increased the use of virtual care, including video appointments as an alternative to in-person consultations, our PAC had not used video consultations for preoperative assessments. A preliminary assessment indicated room for development and improvement of video consultations before routine integration. The initiative focused on enhancing preoperative care without direct patient participation or using identifiable data, potentially offering valuable insights to the broader health care community. This project aimed to develop, implement, and integrate structured steps and process changes using Cisco DX80 Webex devices, measuring the impact on the number or percentage of video consultations through validated continuous quality improvement Plan-Do-Study-Act (PDSA) cycles.

## Methods

### Ethical Considerations

Ethics approval was not obligatory for this initiative; however, we secured Western Research Ethics Board approval (project ID: 118733) before commencing the quality improvement project, conducted between May 2021 and December 2023. No data or personal identifiers from participants were collected. Only information related to the process, such as patient selection, the percentage of successful video consultations, and issues encountered, were documented in a patient-independent manner.

### Study Objective

The primary objective of this study was to develop, implement, and integrate virtual video consultations within the PAC, offering surgical patients the option of a virtual video consultation as an alternative to in-person visits in collaboration with our institution’s multidisciplinary team.

### Participants and Data Sources

Initial data collection covered 4 weeks, from the first to the last day of the month, following the implementation of the March 2021 video consultations. Following the initiation of changes, repeat data were gathered for up to 1 month to evaluate the sustainability and ongoing enhancement of the revised practice. Daily video consultations in each PAC were systematically documented throughout the project to facilitate continuous quality improvement.

In the project’s initial phase, the data supported the suitability of virtual video consultations for patients undergoing bariatric surgery. Our workgroup decided to pilot the project with this population as these patients were already familiar with the Cisco Webex platform. Notably, the acceptance rate for preoperative video consultations among patients undergoing bariatric surgery reached 100% owing to their preexisting use in the bariatric program for preoperative education. This success among this group of patients catalyzed the broader expansion and implementation of video consultations across PACs.

Approximately 100 virtual video consultations were conducted to streamline preoperative video consultation steps. PAC nursing teams held small group meetings to assess the strengths and weaknesses of telephone and video consultations, documenting opinions shared during the discussions. However, no participant-specific information was collected. Stakeholders were briefed on the results of this preliminary assessment.

To identify areas for expansion and improvement, we sought feedback through an audit and a series of PDSA cycles to facilitate change and monitor progress. A key theme emerging from baseline information and staff feedback was enhancing communication between the PAC, patients, and surgeons’ secretaries to offer the option of virtual video consultations postsurgical diagnosis. Additionally, patients’ emails were collected to enable sending invitation links for video consultations. A unique shared mailbox was established for this purpose.

We enlisted champions from each stakeholder group to garner support for our rapid cycle changes. Leveraging data and stakeholder feedback, we used the PDSA methodology to shape our quality improvement strategy over 3 years, abstaining from formal statistical analyses for before-and-after comparisons.

### Strategy

#### Overview

**Figure 1:**
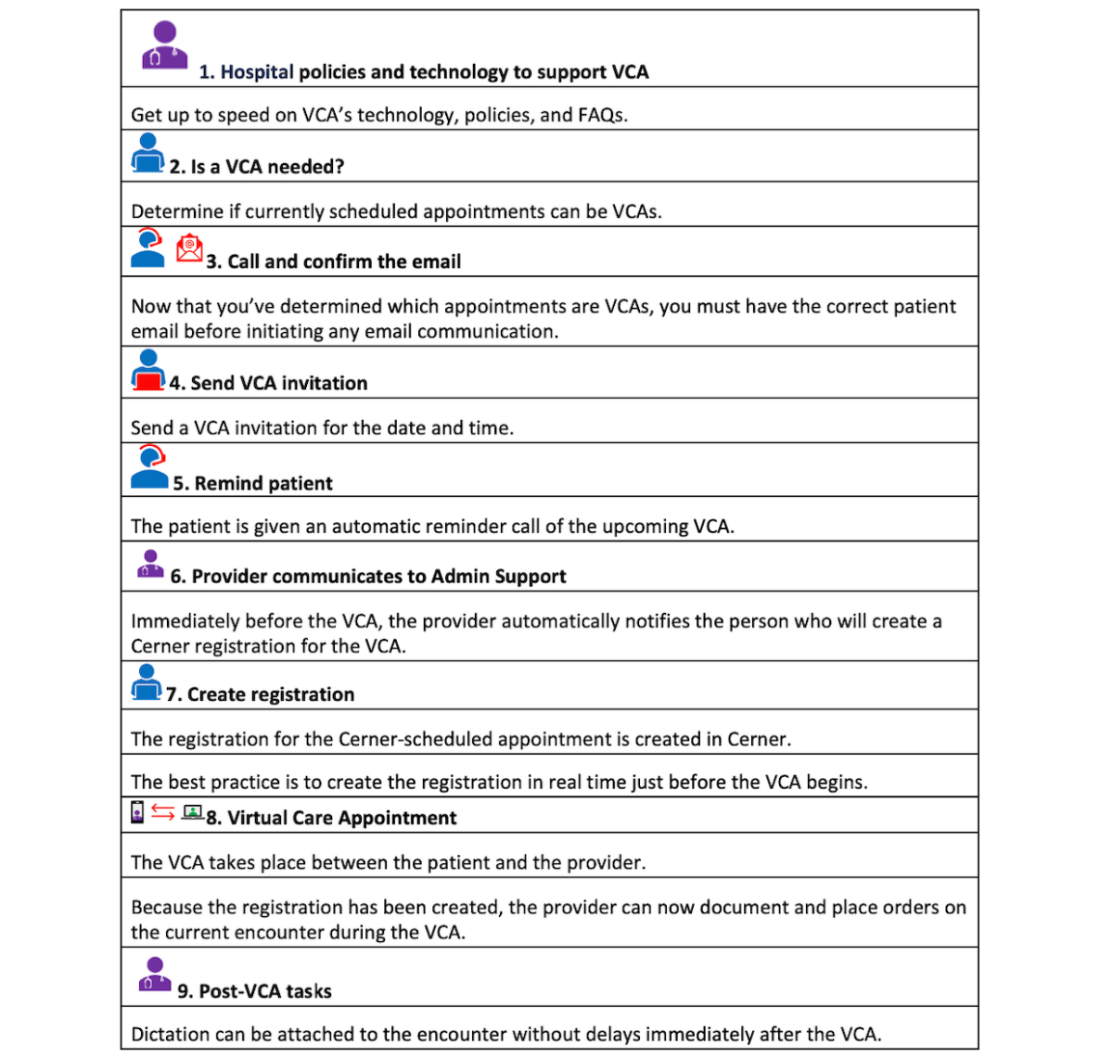
Steps involved in a virtual care appointment (VCA).

We carried out 3 PDSA test cycles over the 3 years. Figure 1 outlines the steps involved in establishing and implementing virtual care appointments.

#### First Intervention: PDSA Cycle 1

Approval from the hospital for the secure Cisco Webex platform prompted the use of cameras for the Cisco DX80 Webex devices in dedicated PAC rooms for video consultations. Collaborative group meetings involving PAC nurses, anesthesiologists, hospital IT staff, and the hospital virtual care team, were held to implement process improvements. Repeated data collection occurred several weeks later using the same preliminary assessment questionnaire after this intervention. The hospital invested in computer-integrated cameras (Cisco DX80 Webex devices) through the virtual care funding program, which were installed in PAC rooms. PAC nurses received 4 training sessions, and video virtual appointment scheduling and registration was established. A common email was created with a shared folder/inbox and regular updates were implemented to enhance virtual care.

#### Second Intervention: PDSA Cycle 2

A dedicated video consultation booking clerk was appointed at the PAC, aiming to boost the percentage of video consultations and simplify the process. Several weeks after the intervention, a reaudit was conducted on the various steps of video consultations.

#### Third Intervention: PDSA Cycle 3

The objective of this stage was to increase the percentage of video consultations further and streamline the process. This involved improving the booking process, routinely collecting patients’ email IDs into electronic records, easing connection to the meeting link (web-based) for patients and health care providers, and integrating them into the patient’s electronic record. With integration of Cisco Webex in Cerner health information technology software, the booking clerk clicks a single button to send the invitation to the patient for a video link. The automatic reminders are sent to the patient to prepare for the video consultation. Once the booking is confirmed, a Webex video link appears in the patient’s electronic chart under the “Virtual Care Appointments” section. The other health care providers can connect with the patient at the scheduled time by clicking the hyperlink “Click here to join.” This prevents clerical errors in sending email invitations and avoids steps for sharing the PINs for the video connections. Training sessions were conducted for the PAC clinic team, including nurses, medicine and anesthesia staff, clinical fellows, and residents. This served as a brief introduction to the initiative and familiarization with the new video consultation process. Changes in provincial rules and regulations for video consultations increased physicians' acceptance rate, addressing persistent improvement opportunities identified in previous implementation cycles.

## Results

**Figure 2:**
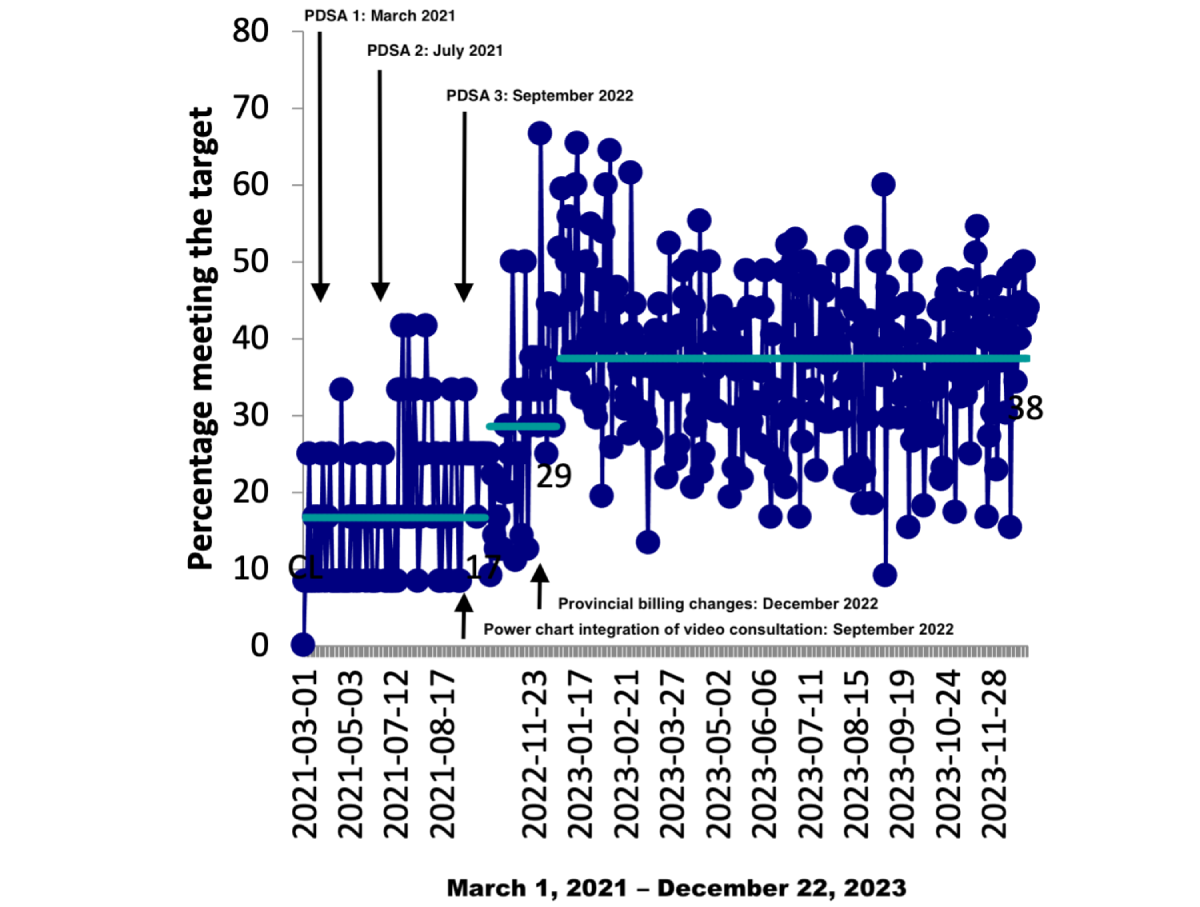
Run chart showing the percentage of patients who completed the video consultation. PDSA: Plan-Do-Study-Act.

Our initial workup indicated that our PAC did not have a video consultation platform before initiating this project. Following the first and second PDSA cycles, the interventions consistently enhanced this metric to a 17% utilization rate, signaling positive developments. As PDSA cycle 3 commenced, there was a substantial increase to a 29% utilization rate during the initial phase. This trend continued, reaching a 38% utilization rate of virtual video consultations in the later phase of the cycle. Utilization was persistently maintained at a high level throughout the entirety of 2023, highlighting the sustained success of our interventions (Figure 2).

**Figure 3:**
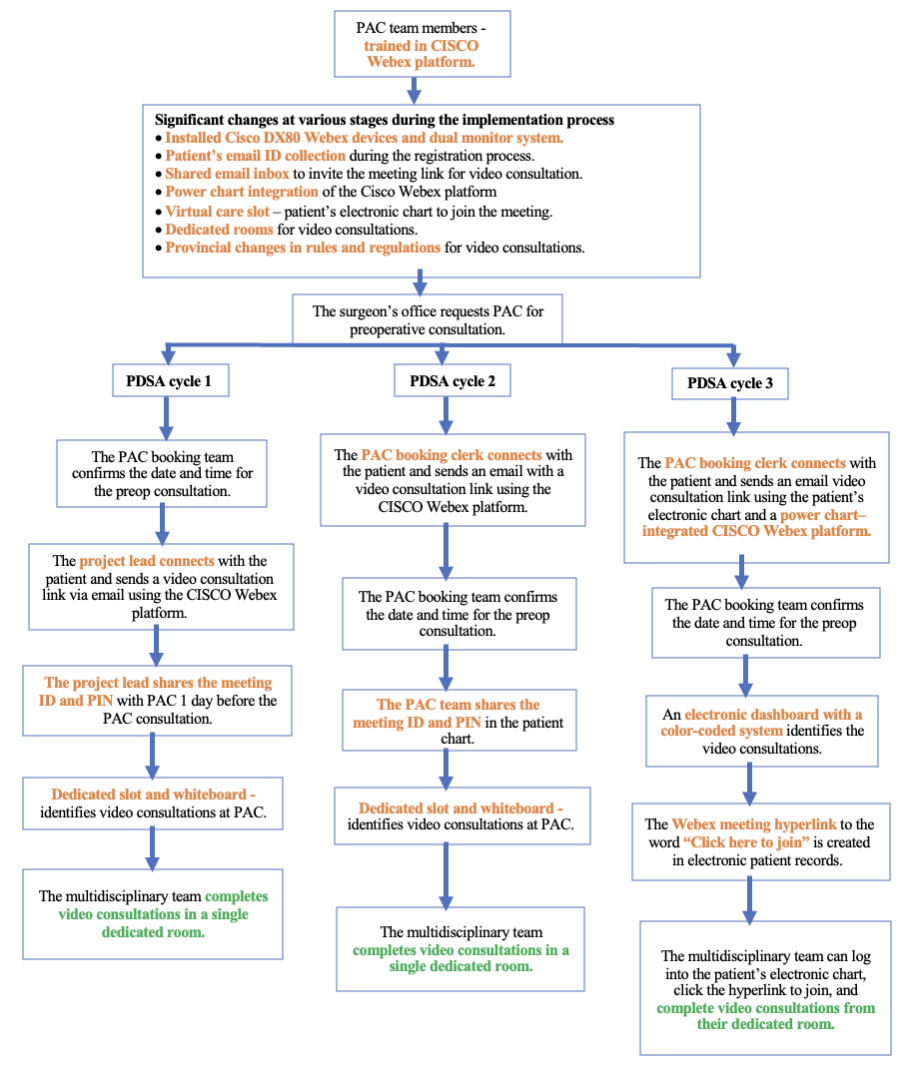
Comprehensive flow diagram detailing the steps and communication pathways involved in patients' video consultations during the PDSA cycles. PAC: preadmission clinic; PDSA: Plan-Do-Study-Act.

Figure 3 provides a comprehensive flow diagram detailing the steps and communication pathways involved in patients’ video consultations. Additionally, this figure highlights the specific changes introduced during the PDSA cycles within the project.

## Discussion

Our results demonstrated that following the interventions through 3 successive PDSA cycles, the utilization rate of video consultations increased to 17% and then to 29% and finally to 38%, maintaining this high level throughout 2023, confirming the sustained success of our quality improvement project.

The PAC under study is part of the perioperative process in a Canadian academic tertiary health sciences center within a publicly funded health care system. While this quality improvement program may have limited applicability to other institutions due to variations in staffing, office space, equipment, technology, expertise, scheduling, communication, patient volumes, and guidelines, the lessons learned here may still offer valuable insights into enhancing patient satisfaction through the introduction of video consultations during the perioperative period of care.

The primary objective of this quality improvement project was to explore, develop, implement, and integrate virtual video consultations within the PAC, ensuring that patient-centered care remains timely, efficient, and safe while preserving the importance of in-person consultations. Key to the project’s success was enhancing communication among PAC staff, patients, and surgeons’ offices; incorporating OneChart Video Webex Appointments; and aligning with provincial changes in rules and regulations. The surgeon’s office electronically communicated patients’ preference for video consultation to the PAC staff while requesting a preoperative consultation. This decentralized the work for the PAC booking clerk. Significant clinical enhancements in video consultations were achieved throughout the preoperative journey without compromising patient care, as evidenced by the increase of video consultations in the PAC from 0% to 38%. The sustainability of said video consultations was confirmed over the past 12 months, indicating enduring improvement and garnering ongoing support and acceptance from the staff. The groundwork for video consultations positions them for long-term continuation, providing a compelling case for improved staffing, IT support, and physical space. This successful implementation of innovative methods empowers stakeholders to advocate for PAC maintenance and further enhancement.

One prominent observation in our project stems from significant variability observed across PACs and within the same clinic on different days, resulting in total virtual video consultation fluctuations. Various factors contribute to this variability, including the volume of patients referred to the PAC from surgical specialties, medical comorbidities of patients rendering them ineligible for video consultations, specific surgical procedures necessitating in-person consultations, variations in the booking staff at the PAC responsible for sending email invitations for video consultations, the number of surgeries conducted during specific slow-down periods such as holidays, and fluctuations in the overall caseload seen in the PAC. Notably, certain days, labeled as “Super Wednesdays” and “Super Tuesdays” in our PAC, presented twice as many patients, leading to increased video consultations on those days. To mitigate the inconsistency in scheduling personnel, a specialized team member was assigned to facilitate clear communication between patients and surgeons’ offices, focusing on effectively organizing video consultations. Some of the other challenges that may be experienced while implementing the video consultations are (1) poor patient internet connectivity, (2) challenges in implementing hardware accessibility in all PAC rooms, and (3) lack of digital literacy among older patients and health care providers.

While the patient information system facilitated data collection, manual data collection remains necessary. Working closely with the hospital’s IT and virtual care teams and their resources proved essential in enhancing patient flow throughout the project by seamlessly integrating video calls into electronic records. In the continuous improvement process, communication options such as “virtual care appointments using Webex” were incorporated into electronic record views, enhancing the efficiency of joining video consultations for the multidisciplinary team in the PAC.

Changes in the PAC were noted during the project, coinciding with broader system and provincial changes. Increased acceptance rates among patients, PAC staff, and physicians led to higher numbers of video consultations. Workforce issues were addressed by assigning additional clerks to assist with the booking process, although no increase in medical and nursing staff occurred. These modifications underscored the clinic’s significance within larger hospitals and the provincial system, emphasizing the need for innovative methods to enhance patient flow, efficiency, and satisfaction without compromising safety.

A key limitation of our study is the lack of consideration for total virtual care usage, as we did not monitor the number of phone visits during the implementation period. Without this information, it is challenging to grasp the impact on overall virtual care usage fully. Another significant limitation is the provincial billing changes that disincentivized phone use, which occurred simultaneously with PDSA cycle 3. These changes substantially affected the PDSA cycle and should be considered when interpreting the results.

Virtual care video appointments offer a reasonable alternative to in-person and phone consultations, gaining prominence during the COVID-19 pandemic and likely continuing to play a significant role in health care [13]. Future directions involve advancing the newly implemented video consultation by integrating an app-based preoperative education system already used at our hospital. Additionally, expanding electronic communication options such as asynchronous preoperative messages will deliver real-time, crucial, and up-to-date information and education about the preoperative journey without interrupting a phone call. This approach aims to empower patients and enhance their compliance with preoperative instructions.

## Abbreviations

LHSC: London Health Sciences Centre
PAC: preadmission clinic
PDSA: Plan-Do-Study-Act
